# Artificial Intelligence in Action: Racial and Gender Disparities in Academic Radiology

**DOI:** 10.7759/cureus.92382

**Published:** 2025-09-15

**Authors:** Lucy Hui, Faisal Khosa

**Affiliations:** 1 Department of Radiology, Faculty of Medicine, University of British Columbia, Vancouver, CAN; 2 Department of Radiology, Vancouver General Hospital, Vancouver, CAN

**Keywords:** academic radiology, faculty advancement, gender and racial inequities, generative artificial intelligence, workforce disparities

## Abstract

Academic radiology continues to face persistent gender and racial disparities in career advancement. The emergence of generative artificial intelligence (AI) platforms offers new opportunities to analyze workforce diversity patterns rapidly and at scale. This study aimed to evaluate the interpretative capacity of three generative AI platforms (i.e., ChatGPT, DeepSeek, and Perplexity) in identifying disparities in academic rank and tenure status across gender and racial subgroups in academic radiology. The outputs of these AI models were compared with conventional human-led analyses for accuracy, limitations, and potential biases.

We prompted each AI model to analyze publicly available American Association of Medical Colleges Faculty Roster data on tenure and rank distribution by gender and race using standardized query templates. Outputs were systematically evaluated for consistency, accuracy, and potential biases against benchmark human-curated studies. Comparative analysis included variations between AI platforms and traditional research methods, with particular attention to how each model interpreted and reported disparities.

The AI models broadly recognized trends in faculty growth and underrepresentation, but interpretations varied. Perplexity and DeepSeek provided more granular insights, such as declining tenure rates and intersectional disparities, while ChatGPT offered less detailed analyses. Key discrepancies included divergent temporal trends and policy recommendations, highlighting AI’s limitations in capturing nuanced sociodemographic patterns.

Generative AI shows promise in analyzing workforce disparities but requires validation to mitigate biases and inconsistencies. When used alongside traditional methods, AI can enhance understanding of inequities in academic medicine, provided that its outputs are critically evaluated for fairness and accuracy.

## Introduction

Disparities in gender and race remain persistent challenges in academic radiology, influencing faculty representation, career advancement, and compensation. Despite progress, women and underrepresented racial and ethnic minorities continue to face significant barriers to attaining higher academic ranks and leadership positions in radiology departments [1]. The intersection of gender and race further compounds these inequities, affecting opportunities for tenure, promotion, and salary equity. As the discipline of radiology strives for greater diversity, equity, and inclusion, there is a critical need to systematically analyze and address these disparities to foster a more representative and equitable academic environment.

Recent studies have documented both encouraging trends and ongoing challenges. For example, the proportion of women in academic radiology faculty and leadership roles has increased over the past decade, yet the representation of women and racial minorities diminishes at senior academic ranks and among department chairs [1,2]. Salary inequities persist, with women and Black faculty earning less than their male and white counterparts, respectively [3]. These patterns reflect broader systemic issues in academic medicine, including implicit bias, unequal access to mentorship, and structural barriers to advancement [4].

The emergence of artificial intelligence (AI) and, specifically, generative AI platforms built on large language models (LLMs), such as ChatGPT, DeepSeek, and Perplexity, has introduced new possibilities for analyzing complex social and organizational phenomena, including disparities in academic medicine [5]. These AI models can rapidly synthesize large datasets, identify patterns, and generate interpretative analyses that may complement or challenge traditional human-led research methods. However, the integration of AI into medical research and practice also raises concerns about the potential for algorithmic bias, which can perpetuate or even exacerbate existing inequities if not carefully identified and mitigated [5].

Bias in AI systems may arise from various sources, including imbalanced training data, flawed model design, or unintentional reinforcement of societal stereotypes [6]. In the context of academic radiology, this means that AI-generated analyses of gender and racial disparities must be scrutinized for validity, transparency, and fairness. Explainable AI methods and fairness-aware systems are increasingly advocated to uncover, understand, and address these biases, ensuring that AI-driven insights contribute to equitable outcomes [7].

Generative AI platforms are now being evaluated for their ability to replicate, augment, or improve upon traditional analyses of workforce diversity, academic rank, and tenure status across different gender and ethnoracial subgroups [8]. Comparing the outputs of multiple AI models with established human-led research offers a unique opportunity to assess the strengths, limitations, and potential biases inherent in AI-driven approaches.

This study aims to compare the interpretative outputs of three leading generative AI platforms (i.e., ChatGPT, DeepSeek, and Perplexity) regarding tenure status and academic rank across five ethnoracial subgroups (White, Asian, Black, Hispanic, and Indigenous) and two gender subgroups (men and women) in academic radiology. By benchmarking these AI analyses against traditional human-led research, we seek to illuminate the validity, limitations, and potential biases of AI in detecting and interpreting gender and racial disparities, ultimately informing best practices for the responsible use of AI in academic medicine.

## Materials and methods

Dataset

We used the publicly available American Association of Medical Colleges (AAMC) Faculty Roster data for the specialty of radiology, spanning the years 1966 to 2021. The dataset included faculty demographic information categorized by academic rank (assistant, associate, full professor) and tenure status (tenured, tenure-track). Individuals were classified by self-reported race (White, Asian, Black, Hispanic, Indigenous) and binary gender (men, women), allowing for intersectional analysis of representation across career stages in academic radiology. The dataset was previously explored by Joarder et al. using standard statistical techniques and thematic analysis [9].

AI tools and prompt design

We evaluated three state-of-the-art generative AI platforms - ChatGPT GPT-4, DeepSeek V3, and Perplexity Sonar - using their most current publicly available versions as of June 2025. Given that AI model outputs are known to vary with prompt structure, we employed a single standardized prompt across all platforms to allow for direct comparison [10]. Each platform received an identical structured prompt: "Perform an analysis of this dataset and report any notable trends, patterns, or insights you identify. Analyze all the sheets in this Excel. Provide a descriptive summary of key findings and statistically significant results." To ensure comparability across platforms, we employed a single standardized query template rather than tailoring prompts to individual models. This approach was designed to reflect the type of straightforward, surface-level query that an academic researcher without advanced prompt-engineering expertise might use, thereby allowing us to assess each model’s baseline interpretative capacity under consistent conditions. All AI models were accessed through publicly available interfaces, and no model had prior exposure to the dataset used in this study. This approach allowed assessment of baseline interpretative capacity and facilitated direct comparison between platforms, while minimizing potential bias introduced by model-specific fine-tuning or prior task-specific learning. Furthermore, the AI tools were provided with direct access to the dataset but were deliberately not given the published article to prevent potential bias from existing human analyses. This standardized approach allowed us to assess each model's native analytical capabilities while maintaining consistency across platforms. All queries were executed within 24 hours to ensure version consistency and minimize potential temporal variations in model outputs. The prompt design intentionally avoided leading questions to evaluate how each AI would independently identify and prioritize findings from the raw data.

Analysis

Outputs from three generative AI models were compared against the findings reported by Joarder et al. across 11 key themes: overall faculty growth, tenured faculty trends, on-track faculty trends, not on-track faculty trends, professor rank trends, associate professor rank, assistant professor rank, gender diversity, racial/ethnic diversity, key insights, and recommendations [9]. For each theme, the AI-generated outputs were evaluated for consistency with the published data, and any discrepancies, omissions, or errors were carefully documented. To maintain the integrity of the AI interpretations and avoid introducing human bias, no manual corrections or adjustments were made to the AI outputs. This approach enabled an objective assessment of the capabilities and limitations of generative AI in replicating and interpreting complex demographic analyses in academic radiology, while also highlighting areas where AI models may diverge from established findings or overlook critical nuances.

## Results

A comprehensive comparison of the three AI-generated outputs with the findings of Joarder et al.’s study is presented in Table 1 [9]. The original output from all three AI models can be found in the Appendix.

**Table 1:** Comparative summary of AI output by thematic domain

Theme	ChatGPT Findings	Perplexity Findings	DeepSeek Findings	Joarder et al.'s Findings
Overall faculty growth	13-fold increase; steepest growth in 1990; peaked in 2020	Grew from 759 to 10,175; steep growth in 1970s and 1990s; plateau since 2017	13.4-fold increase; steepest growth in 1970-1980 and 2000-2010; plateau recently	Grew from 759 to 10,175
Tenured faculty trends	Significant growth from 1966 to 1995 and plateau recently; peaked in 1995; increase in women tenured faculty	Peaked in 1990s and decline in 2021; White faculty remain majority but decreased; Asian, Black, Hispanic, Indigenous faculty increased, especially for women but still low	Increased from 1966 to 2021 but growth has declined in recent years; gender disparity noted, but improving; Asian faculty saw most growth, Black and Hispanic faculty low, and Indigenous faculty nearly nonexistent	Significantly more White men in tenured position than all other groups throughout the study period; Asian men had largest increase; no significant changes in representation seen among Black men over the study period
On-track faculty trends	Not detailed	Increased from 1966 to 2021; Asian and White faculty dominant but all underrepresented groups have seen gradual increase; women’s representation has grown	Increased from 1966 to 2021; women’s representation improved, especially among Asian and White	White men significantly decreased over time; Asian men had largest significant increase in representation
Not on-track faculty trends	Not detailed	Major growth from 1966 to 2021; implies the majority of positions are non-tenure-track; all groups have increased; women’s representation higher in non-tenure tracks than tenure tracks	Grew from 1966 to 2021; women are minority but numbers have increased; White faculty dominant but diversity has improved slightly over time	Not detailed
Professor rank trends	Substantial increase from 1966 to 2021; women increased but still underrepresented.	Growth from 1966 to 2021; predominantly White men but all groups have increased slowly; women remain minority, increase slowly	Dominated by White men	Dominated by White men, similar rate of change in representation; increasing representation of Asian men and women; no significant changes in representation among Black, Hispanic, and Indigenous men
Associate professor rank	Not detailed	Growth from 1966 to 2021; increases in all groups, more pronounced rise in Asian and Hispanic faculty; women’s numbers have increased, among White and Asian faculty	More balanced gender distribution than professors but skewed towards men; Asian faculty saw significant growth	Dominated by White men, similar rate of change in representation; no significant changes in representation among Black, Hispanic, and Indigenous men
Assistant professor rank	Not detailed	Growth from 1966 to 2021; greatest gains in Asian and Hispanic faculty, both men and women; women’s representation is highest at this rank	Highest growth in diversity, with more women; Black and Hispanic faculty are better represented at this level but still lag behind White and Asian faculty	Dominated by White men, similar rate of change in representation; no significant changes in representation among Black, Hispanic, and Indigenous men
Gender diversity	Not specifically detailed	Women’s share has growth at all rank and tracks, especially in non-tenure-track and assistant professor roles; women remain underrepresented at professor and tenured levels	Gender gap persists; leaky pipeline effect is evident: women are better represented at assistant professor levels	The proportion of White women reached a peak in mid-1990s after which it began to decline
Racial/ethnic diversity	Not specifically detailed	Asian faculty have seen significant growth among assistant professors and non-tenure-track role; Black, Hispanic, Indigenous faculty remain underrepresented	White faculty still dominate but Asian faculty have seen growth; Black, Hispanic, and Indigenous faculty remain severely underrepresented at all levels	White faculty consistently highest represented ethnoracial-gender group; minority groups collectively comprise less than 5% of academic radiology
Key insights	Expansion of radiology over time; plateau in tenured position vs continued growth in total radiologist points towards more clinical or adjunct appointments; slow progress in gender parity	Tenure is declining; diversity is improving; largest gains in diversity are at assistant professor and non-tenure-track levels	Recent declines in tenured faculty, possibly due to shifting academic structures; diversity progress is slow	Not specifically detailed
Recommendations	Not detailed	Support career advancement; monitor non-tenure-track growth; expand recruitment and retention efforts for Black, Hispanic, Indigenous faculty	Targeted recruitment and retention; mentorship programs; track progress	Policy-makers should direct their focus on creating environments conducive to equal opportunity for women

The comparative analysis of AI-generated outputs (ChatGPT, Perplexity, DeepSeek) against Joarder et al.'s findings revealed both consistencies and discrepancies across key themes [9]. Overall faculty growth was broadly aligned, with all AI models recognizing a substantial increase in academic radiology faculty from 759 to 10,175 over the study period. However, while Joarder et al. did not specify peak growth decades, ChatGPT emphasized the 1990s, Perplexity highlighted the 1970s and 1990s, and DeepSeek noted growth surges in the 1970-1980 and 2000-2010 periods [9]. All models acknowledged a recent plateau, though only Perplexity and DeepSeek explicitly linked it to post-2017 trends, whereas Joarder et al. did not address this stagnation [9].

Tenured faculty trends showed notable divergence. ChatGPT reported significant growth until 1995, followed by a plateau, while Perplexity and DeepSeek noted declines in recent years. This was a trend not explicitly discussed by Joarder et al. All AI models highlighted persistent gender and racial disparities, with White men dominating tenured positions. However, Perplexity and DeepSeek provided more granular insights, such as Asian faculty experiencing growth, and Black, Hispanic, and Indigenous faculty remaining severely underrepresented. Joarder et al. corroborated the dominance of White men but did not detail declines in tenure rates or recent demographic shifts [9].

Rank-specific trends further illustrated variability. For professor-rank faculty, all AI models agreed on White male dominance, but only Perplexity and DeepSeek noted slow increases in women and Asian faculty representation, aligning partially with Joarder et al.’s observation of rising Asian male representation [9]. At the assistant professor level, Perplexity and DeepSeek emphasized greater gender and racial diversity, particularly for women and Asian/Hispanic faculty, whereas Joarder et al. reported no significant changes for Black, Hispanic, or Indigenous men [9]. Gender and racial diversity findings were mixed: while Perplexity and DeepSeek identified progress in non-tenure-track and assistant professor roles, ChatGPT omitted specifics, and Joarder et al. underscored stagnant representation for minority groups overall [9].

Key insights and recommendations diverged significantly. ChatGPT noted slow progress in gender parity, while Perplexity and DeepSeek highlighted declining tenure rates and leaky pipeline effects. Joarder et al. did not explicitly discuss these structural shifts [9]. Recommendations from Perplexity and DeepSeek were more actionable, emphasizing mentorship and targeted recruitment for underrepresented groups, whereas Joarder et al. broadly advocated for policy-level interventions. These discrepancies suggest that while AI models capture overarching trends, their interpretations of nuances, particularly regarding temporal shifts and policy implications, vary considerably.

## Discussion

The comparative analysis of three generative AI models (i.e., ChatGPT, Perplexity, and DeepSeek) revealed significant variations in their ability to interpret and report on gender and racial disparities in academic radiology. While all models identified broad trends, such as the exponential growth of faculty and the decline in tenured positions overall, their depth of analysis and thematic coverage differed markedly. ChatGPT provided the least comprehensive assessment, omitting critical discussions on ethnoracial diversity and failing to analyze rank-specific trends for associate and assistant professors, as well as on-track and off-track faculty. Its outputs were largely descriptive, focusing instead on visual data representations, such as line graphs depicting overall faculty growth. This limitation suggests that ChatGPT may prioritize high-level summaries over nuanced, intersectional analyses. This is a potential drawback when assessing complex sociodemographic disparities. This finding is in contrast to a study analyzing ChatGPT’s ability to perform statistical analysis, which found that it was able to accurately perform data processing, categorization, and tabulation [11]. However, in this study, specific prompts were needed to guide ChatGPT through each step of analysis unlike the broad prompts used in our study [11,12]. Indeed, it has been shown that when provided generic prompts, ChatGPT-generated responses were low in quality [13]. In contrast, Perplexity and DeepSeek delivered more balanced and detailed examinations, systematically addressing gender and ethnoracial representation across all academic ranks and tenure categories. Recent literature suggests that DeepSeek achieved the highest accuracy rate in answering medical questions, followed by ChatGPT-4o and Perplexity [14]. Their ability to identify and contextualize disparities, such as the underrepresentation of Black, Hispanic, and Indigenous faculty, demonstrates a stronger capacity for replicating the granularity of human-led research.

A notable divergence between the AI models and Joarder et al. was their interpretation of structural trends in academic radiology [9]. All three AI platforms independently highlighted the decline in tenured faculty and speculated about systemic shifts, such as the move toward non-tenure-track appointments. This is a finding not assessed in Joarder et al.'s analysis [9]. This suggests that AI models may detect latent patterns in large datasets that conventional methodologies may overlook, potentially serving as a tool for hypothesis generation in future research [15]. This suggests a potential role for AI models in identifying gaps in consideration and informing more inclusive research design and interpretation. However, the absence of statistical validation across all AI outputs raises concerns about the reliability of their claims. For instance, while Perplexity and DeepSeek noted "significant growth" among Asian faculty or women in assistant professor roles, neither model applied statistical tests to confirm these observations. Generative AI are prone to provide confident responses that are non-accurate to the question [16]. Without such rigor, their conclusions risk overstating trends or misrepresenting marginal changes as substantive progress, particularly as all three models are non-explainable AI.

Another key distinction was the unsolicited inclusion of recommendations by Perplexity and DeepSeek, which proposed interventions such as targeted recruitment and mentorship programs for underrepresented groups. While these suggestions align with established diversity initiatives, their spontaneous generation raises questions about the models’ underlying design. Are these recommendations derived from the data or do they reflect embedded biases or generalizations in the AI’s training? Conversely, ChatGPT’s avoidance of prescriptive solutions may indicate a more conservative approach to data interpretation. The variability in outputs highlights the importance of transparency in how AI models prioritize and synthesize information, particularly when applied to sensitive domains such as equity research.

The findings highlight both the potential and limitations of generative AI in disparities research. On one hand, models such as Perplexity and DeepSeek demonstrate an ability to parse complex demographic data and identify understudied trends, offering a scalable complement to traditional analysis. On the other hand, AI’s occasional omissions emphasize the need for human oversight. It is also important to note that prompt design strongly influences the depth and specificity of LLM outputs. While we used a single standardized prompt for comparability, iterative refinement or inclusion of instructions to identify potential sources of bias could yield more detailed and nuanced analyses. Future work should explore hybrid frameworks that pair AI’s analytical speed with researchers’ expertise to validate findings and contextualize results. Additionally, improving prompt specificity, such as explicitly requesting statistical tests or intersectional analysis, could reduce variability in AI outputs. Finally, addressing algorithmic bias through targeted training on diverse datasets and fairness-aware model design will be critical to ensure that AI tools advance, rather than inadvertently perpetuate, equity goals in academic medicine.

Limitations

Several limitations should be acknowledged in this study. First, the use of generative AI tools such as ChatGPT, DeepSeek, and Perplexity AI relies heavily on the models’ training data, architecture, and internal reasoning mechanisms, which are not transparent or fully interpretable. As such, any insights or omissions in the AI-generated analyses may reflect underlying model biases or limitations rather than deficiencies in the dataset itself. Second, while efforts were made to standardize the prompt and ensure version consistency, AI model behavior may still vary subtly due to stochasticity in output generation, potentially affecting reproducibility. Third, the exclusion of Joarder et al.'s paper from the AI’s input was intentional to preserve analytical independence, but it also meant that the models lacked the benefit of human contextualization, which may have limited their ability to interpret certain sociocultural or historical nuances in the data. Lastly, our study used a single standardized prompt to evaluate interpretative capacity across models. While this design enhanced reproducibility and comparability, it did not optimize for maximal model performance and limits generalizability to alternative prompting strategies. Alternative strategies, such as iterative prompting or multi-step scaffolding, may yield more detailed outputs and represent an important avenue for future research.

Future implications

The findings of this study highlight several promising directions for leveraging AI in diversity research while addressing current limitations. Future work should focus on developing hybrid frameworks that combine AI’s efficiency in pattern detection with human expertise for statistical validation and contextual interpretation. Fine-tuning AI models on intersectional datasets and incorporating fairness-aware algorithms could mitigate biases and improve the accuracy of disparity analyses. Additionally, refining prompt engineering to explicitly request statistical testing and granular demographic breakdowns may enhance the consistency and depth of AI-generated insights. The ability of AI models to identify understudied trends, such as structural shifts in tenure practices, also suggests their potential as hypothesis-generating tools to guide targeted research. Ultimately, integrating AI as a complementary tool in equity research, rather than a standalone solution, could accelerate progress toward more inclusive academic environments while ensuring rigorous, actionable findings.

## Conclusions

This study highlights the potential of generative AI platforms to analyze gender and racial disparities in academic radiology, while also underscoring their limitations and variability. The comparative analysis revealed that while ChatGPT, DeepSeek, and Perplexity broadly aligned with human-led research on faculty growth and representation trends, their interpretations of nuanced patterns, such as temporal shifts in tenure rates and intersectional disparities, diverged significantly. Notably, the AI models varied in the depth and specificity of their outputs, with some models generating more detailed insights and recommendations than others; these differences may reflect interactions between the prompt and model architecture rather than inherent capabilities of any specific platform. These discrepancies emphasize the need for rigorous validation of AI-generated findings, particularly in sociodemographic research where biases can perpetuate inequities. Moving forward, generative AI should be used as a complementary tool alongside traditional methods, with careful attention to algorithmic transparency and fairness. By addressing these challenges, AI can contribute meaningfully to advancing diversity, equity, and inclusion in academic medicine.

## Figures and Tables

**Figure 1:**
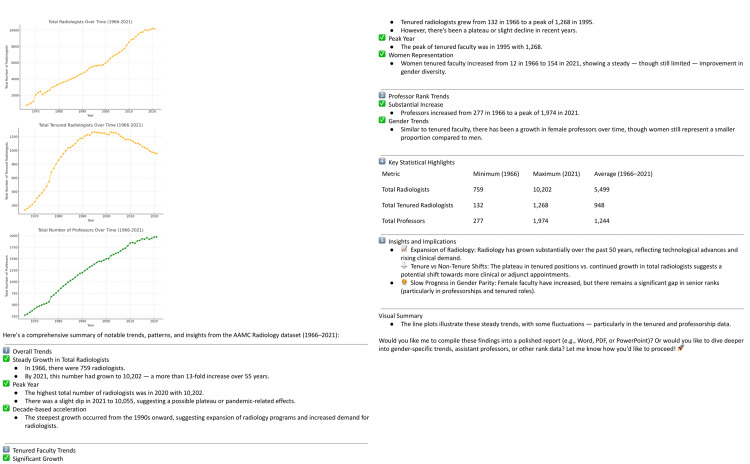
ChatGPT output on June 5, 2025

**Figure 2:**
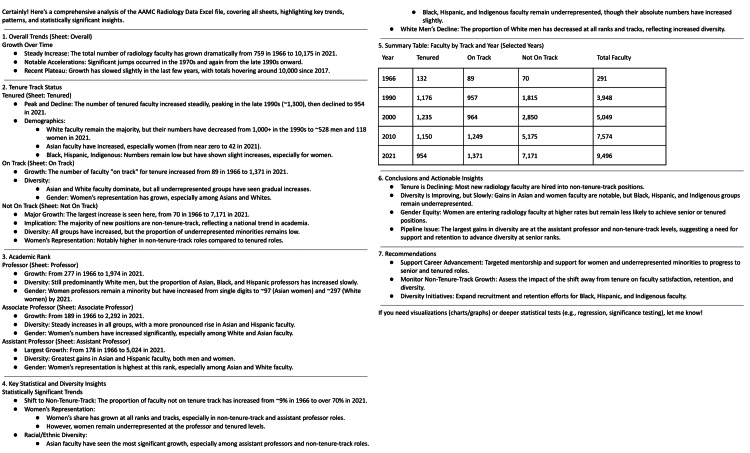
Perplexity output on June 5, 2025

**Figure 3:**
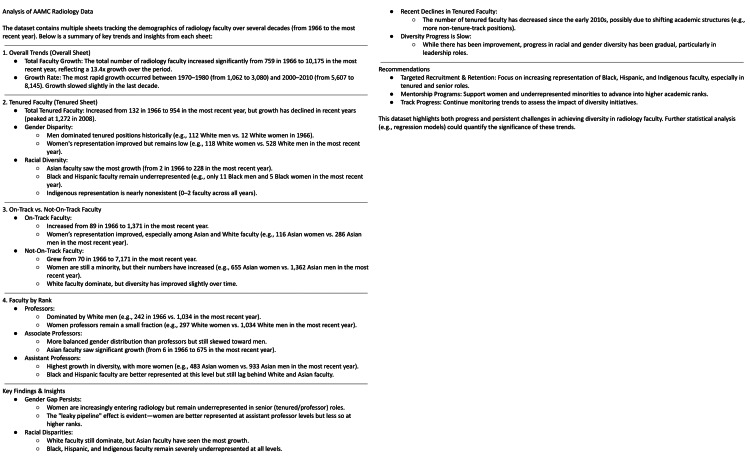
DeepSeek output on June 5, 2025

## References

[1] Sharma S, Malik A, Matschek J, Zaki-Metias KM, Gandhi R, Yong-Hing CJ, Khosa F (2025). Assessing and improving women representation in radiology leadership positions. Curr Probl Diagn Radiol.

[2] Schilling SM, Trout AT, Ayyala RS (2023). Gender disparity in academic advancement: exploring differences among adult and pediatric radiologists. Pediatr Radiol.

[3] Malhotra A, Futela D, Joshi R (2024). Academic radiology physician financial compensation in the United States: trends and distribution. Radiology.

[4] Yuen J, Kulathaivelu R, Hussain M (2023). Gender differences in academic rank, leadership, and awards among NIH grant recipients in diagnostic radiology. J Womens Health (Larchmt).

[5] Green BL, Murphy A, Robinson E (2024). Accelerating health disparities research with artificial intelligence. Front Digit Health.

[6] Celi LA, Cellini J, Charpignon ML (2022). Sources of bias in artificial intelligence that perpetuate healthcare disparities-a global review. PLOS Digit Health.

[7] Ferrara C, Sellitto G, Ferrucci F, Palomba F, De Lucia A (2024). Fairness-aware machine learning engineering: how far are we?. Empir Softw Eng.

[8] Mathis MS, Badewa TE, Obiarinze RN, Wilkinson LT, Martin CA (2021). A novel use of artificial intelligence to examine diversity and hospital performance. J Surg Res.

[9] Joarder I, Ahmadi S, Ding J, Khosa F (2025). Gender and race in radiology: an intersectional analysis of the American Association of Medical Colleges (AAMC) database from 1966 to 2021. Curr Probl Diagn Radiol.

[10] Sarangi PK, Mondal H (2024). Response generated by large language models depends on the structure of the prompt. Indian J Radiol Imaging.

[11] Ruta MR, Gaidici T, Irwin C, Lifshitz J (2025). ChatGPT for univariate statistics: validation of AI-assisted data analysis in healthcare research. J Med Internet Res.

[12] Ordak M (2023). ChatGPT's skills in statistical analysis using the example of allergology: do we have reason for concern?. Healthcare (Basel).

[13] Cheng SL, Tsai SJ, Bai YM (2023). Comparisons of quality, correctness, and similarity between ChatGPT-generated and human-written abstracts for basic research: cross-sectional study. J Med Internet Res.

[14] Bashah A, Salem A, Al-Waqeerah A (2025). Evaluation of DeepSeek, Gemini, ChatGPT-4o, and Perplexity in responding to salivary gland cancer. BMC Oral Health.

[15] Lehr SA, Caliskan A, Liyanage S, Banaji MR (2024). ChatGPT as research scientist: probing GPT's capabilities as a research librarian, research ethicist, data generator, and data predictor. Proc Natl Acad Sci U S A.

[16] Alkaissi H, McFarlane SI (2023). Artificial hallucinations in chatGPT: implications in scientific writing. Cureus.

